# Parkinson disease polygenic risk score is associated with Parkinson disease status and age at onset but not with alpha-synuclein cerebrospinal fluid levels

**DOI:** 10.1186/s12883-017-0978-z

**Published:** 2017-11-15

**Authors:** Laura Ibanez, Umber Dube, Benjamin Saef, John Budde, Kathleen Black, Alexandra Medvedeva, Jorge L. Del-Aguila, Albert A. Davis, Joel S. Perlmutter, Oscar Harari, Bruno A. Benitez, Carlos Cruchaga

**Affiliations:** 1 Department of Psychiatry, School of Medicine, Washington University in Saint Louis, Saint Louis, MO USA; 2 Hope Center Program on Protein Aggregation and Neurodegeneration, Washington University in Saint Louis, Saint Louis, MO USA; 3 Medical Scientist Training Program, School of Medicine, Washington University in Saint Louis, Saint Louis, MO USA; 4 Department of Neurology, School of Medicine, Washington University in St Louis, Saint Louis, MO USA; 5 Department of Medicine, School of Medicine, Washington University in Saint Louis, Saint Louis, MO USA

**Keywords:** Parkinson disease, Genetics, Age at onset, Biomarkers, Polygenic risk score

## Abstract

**Background:**

The genetic architecture of Parkinson’s Disease (PD) is complex and not completely understood. Multiple genetic studies to date have identified multiple causal genes and risk loci. Nevertheless, most of the expected genetic heritability remains unexplained. Polygenic risk scores (PRS) may provide greater statistical power and inform about the genetic architecture of multiple phenotypes. The aim of this study was to test the association between PRS and PD risk, age at onset and cerebrospinal fluid (CSF) biomarkers (α-synuclein, Aβ_1–42_, t-tau and p-tau).

**Methods:**

The weighted PRS was created using the genome-wide loci from Nalls et al., 2014 PD GWAs meta-analysis. The PRS was tested for association with PD status, age at onset and CSF biomarker levels in 829 cases and 432 controls of European ancestry.

**Results:**

The PRS was associated with PD status (*p* = 5.83×10^−08^) and age at onset (*p* = 5.70×10^−07^). The CSF t-tau levels showed a nominal association with the PRS (*p* = 0.02). However, CSF α-synuclein, amyloid beta and phosphorylated tau were not found to be associated with the PRS.

**Conclusion:**

Our study suggests that there is an overlap in the genetic architecture of PD risk and onset, although the different loci present different weights for those phenotypes. In our dataset we found a marginal association of the PRS with CSF t-tau but not with α-synuclein CSF levels, suggesting that the genetic architecture for the CSF biomarker levels is different from that of PD risk.

**Electronic supplementary material:**

The online version of this article (10.1186/s12883-017-0978-z) contains supplementary material, which is available to authorized users.

## Background

Parkinson’s Disease (PD) is the second most common neurodegenerative disorder, after Alzheimer’s Disease (AD) [1]. PD is a slowly progressive chronic neurodegenerative disorder characterized by motor symptoms such as resting tremor, bradykinesia, rigidity and postural instability among others [2]. The accumulation of Lewy bodies formed by α-synuclein deposits [2, 3] and loss of dopamine neurons in the substantia nigra are key pathologic findings. The prevalence of PD varies with age, from one to two percent at ages of 55–65 years to 3.5% at 85–89 years, but the underlying cause of most cases of PD remains unknown [1].

The genetic architecture of PD is complex and not completely understood. Several genetic studies have identified multiple causative genes as well as common and rare variants. Initial studies focused on early-onset and familial PD discovered rare mutations in 16 loci (*PARK1* to *20)*, also known as the “Mendelian PD genes”. The reported variants in those genes have large effect sizes, meaning a high probability of developing PD [4, 5]. More recently, genome-wide association studies (GWAs) have found 26 PD risk loci with relatively small effect size [6, 7]. However, these PD loci only explain six to seven percent of the phenotypic variability and three to five percent of the genetic variability associated with PD [8]. Thus, despite the great number of genetic studies, a significant proportion of the genetic contribution in PD remains to be described. Although the amount of phenotypical variability explained by these GWAS hits is low, multiple studies indicate that the Polygenic Risk Scores (PRS) capture the overall genetic architecture of complex traits [9–12]. PRS aggregates the effects of multiple genetic markers (both protective and risk variants) and can be used to evaluate the potential overlap in the genetic architecture of different complex traits, or different phenotypes for the same complex traits [13].

PD diagnosis can be difficult due to overlapping clinical manifestations of multiple parkinsonian syndromes and the lack of a specific biomarker. Cerebrospinal fluid (CSF) levels of alpha synuclein (α-syn) have emerged as one of the most promising biochemical biomarkers, but its informative value is not sufficient to be used as a diagnostic tool [14, 15]. Duplications and mutations of the alpha-*synuclein* gene (*SNCA)* have been found in familial PD, but the role of α-syn and the functional consequences of the mutations are still to be characterized. Although Lewy bodies primarily composed of α-syn, they also contain tau. In fact, the continuum theory postulates that α-syn and tau interaction is central to neurodegeneration [16]. This theory is supported by the pathological overlap between tauopathies such as progressive supranuclear palsy (PSP), corticobasal degeneration (CBD) and synucleinopathies such as PD [17, 18]. Moreover, recent studies have shown that CSF levels of α-syn, total tau (t-tau) and phosphorylated tau (p-tau) and probably amyloid-beta 1–42 (Aβ_1–42_) are significantly lower in PD individuals compared to healthy controls [14, 19]. However, studies looking at the overlap between the genetic architecture of PD risk and biomarker levels have yet to be carried out.

Polygenic Risk Scores (PRS) have been successfully used to capture the additive effect of common variants in order to calculate the overall risk of an individual or to identify individuals at risk [20]. Even though the predictive power and accuracy of PRS are still insufficient to be applied in a clinical setting [20, 21], PRS are becoming more informative with larger GWAs and increasing numbers of GWAs hits. For example, the first schizophrenia PRS explained three percent of the variance, but the recently published version explained 18% [22–24]. Previous attempts to create a PRS for PD were unsuccessful at predicting risk of PD, but correlated with age at onset [10]. Others have been successful in the prediction of PD, but have not yet been correlated with other characteristics of the disease or possible disease biomarkers [25]. In an attempt to address this issue, we created a PRS from PD risk from a recent meta-analysis that included 13,708 PD cases and 95,282 controls [7] and tested for association with PD risk, age at onset and CSF biomarkers (α-syn, Aβ_1–42_, t-tau and p-tau).

## Methods

### Sample description

This study was performed using samples from individuals with European ancestry from two PD datasets: the Parkinson’s Progression Markers Initiative (PPMI) and the Washington University in Saint Louis (WUSTL) Movement Disorder Center (Table 1). All individuals carrying pathogenic mutations in *LRRK2*, *DJ1*, *PARK2 or PINK1* genes, duplications in the *SNCA* gene or risk-associated variants in the *TREM2*, *GBA* or *MAPT* genes [26, 27] were excluded from these analyses. WUSTL PD samples were also screened for the presence of Hexanucleotide expansions in the *C9ORF72* gene [28]. Written informed consent was obtained from all participants prior to their enrollment. This study was approved by the Washington University in Saint Louis Institutional Review Board (approval number: 201107095). PD clinical diagnoses were based on UK Brain Bank criteria [29]. Demographic characteristics of the full cohorts have been published for the PPMI and the WUSTL datasets [26, 30, 31]. Briefly, the PPMI individuals selected for this study were 336 cases and 139 controls with European ancestry, 34.38% being female. The WUSTL selection was comprised of 493 cases and 293 controls with European ancestry, 41.61% being female. Age at onset for PD cases and age at last assessment for controls was available for all individuals.

**Table 1:** Demographic characteristics of the studied population

	Combined		PPMI		WUSTL	
	Controls (432)	Cases (829)	Controls (139)	Cases (336)	Controls (293)	Cases (493)
Females, n (%)	197 (45.60)	278 (33.53)	94 (67.63)	116 (34.52)	166 (56.66)	162 (32.86)
Age at Onset (years)	–	60.99	–	62.50	–	61.00
Age at Last Assessment (years)	63.60	64.70	61.30	61.74	64.86	67.40
Age at Lumbar Puncture (years)	62.73	63.61	63.04	63.07	60.60	65.65
Family History of PD, n (%)	11 (2.55)	211 (25.45)	8 (5.76)	79 (23.51)	3 (1.02)	132 (26.77)
	Controls (155)	Cases (422)	Controls (135)	Cases (334)	Controls (20)	Cases (88)
α-syn (pg/mL ± SD)	829.30 ± 853.67	1800.90 ± 706.85	2173.5 ± 800.80	1865 ± 717.65	1725 ± 453.77	1556.20 ± 608.45
Aβ_1–42_ (pg/mL ± SD)	−0.004 ± 0.12^a^	−0.013 ± 0.12^a^	378.40 ± 104.37	377.30 ± 99.63	926.40 ± 148.02	808.30 ± 215.07
t-tau (pg/mL ± SD)	0.021 ± 0.18^a^	−0.038 ± 0.16^a^	53.95 ± 22.90	46.18 ± 18.77	250.40 ± 113.58	222.87 ± 85.34
p-tau (pg/mL ± SD)	−0.004 ± 0.21^a^	−0.057 ± 0.21^a^	17.49 ± 9.49	15.95 ± 9.40	47.00 ± 17.51	37.19 ± 13.23

^a^Due to methodological differences the raw values cannot be combined. The normalized and standardized values are shown

CSF biomarker levels were available for 422 PD cases and 155 controls (Table 1). Of those, 469 were from the PPMI cohort (334 cases and 135 controls) and 108 were from the WUSTL cohort (88 cases and 20 controls). In each cohort the CSF biomarkers were quantified using different kits. The PPMI study measured Aβ_1–42_, t-tau and p-tau using the xMAP-Luminex platform with INNOBIA AlzBio3 immunoassay kit-based reagents (Fujirebio-Innogenetics, Ghent, Belgium) and α-syn with a commercial ELISA kit (Covance, Dedham, MA) [32]. The WUSTL cohort used the INNOTEST assay to test Aβ_1–42_, t-tau and p-tau, and the same kit as PPMI was used to measure α-syn levels [19]. Due to methodologic differences, and prior to data combination, the raw CSF biomarker level values were normalized (log_10_-transformed) and standardized using the mean of each dataset to perform the joint analyses.

The study had 90% power (considering α = 0.05, two sided) to capture the effect if the overall minor allele frequency (MAF) of the PRS was 5%. With a total sample size of 1261 individuals and overall MAF for the PRS of 30% we had the necessary power to detect differences in the mean PRS between cases and controls.

### Calculation of the polygenic risk score

Both the PPMI and WUSTL datasets are available by request from the PPMI website (www.ppmi-info.org) and the corresponding author of this manuscript respectively. Both populations were genotyped using the Illumina ImmunoChip and *NeuroX* (240,000 variants corresponding to exome content and 24,000 variants focusing on neurodegenerative diseases [33]). A subset of the WUSTL dataset was genotyped with the HumanCoreExome (*N* = 38). Both datasets were imputed using SHAPEIT/IMPUTE2 [34, 35] with the 1000 Genomes Project as the reference panel [36]. All genotypes with dosage levels <0.9 for all three possible genotypes or with information scores <0.3 were excluded. Variants out of Hardy Weinberg Equilibrium (HWE) (*p* < 1×10^−06^) or with a genotyping rate below 95% were removed. The different arrays were imputed separately and then combined. We only then analyzed those variants that had an overall call rate in the joint-imputed file of 85%.

Population structure was inferred by principal component (PC) analysis using PLINK v.1.9 [37]. Only individuals that clustered with the European-American cluster were included for the analysis.

The PRS was computed using the binary logarithm transformation of the reported ORs [7]. We had no access to full summary statistics for the meta-analysis to calculate a PRS as described by the Schizophrenia Consortia. Therefore, we created a PRS using only the genome wide loci associated with PD risk in the most recent meta-analysis that included 13,708 PD cases and 95,282 controls [7]. The genotyping rate for all the genome wide loci to be included in the PRS was calculated (Table 2). Sixteen out of twenty-six variants had an overall call rate (genotyped or imputed) of >85% (mean genotype call rate across the three platform used for genotyping) and were included in the PRS. For the other ten variants, we attempted to select a genetic proxy with an overall call rate > 85% that was in linkage disequilibrium (R2 > 0.90) with the reported GWAs hit. Unfortunately, no suitable proxies were found. Thus, the final PRS value included 16 variants (Table 2) and was computed using PLINK 1.9 [37].

**Table 2:** PD Genetic Risk Score Variants

Variant	Chr	Position	Gene or Nearest Genes	Effect Allele	Known Loci			Current Study		GRS Weight
					MAF	P.Value	OR	MAF	Call Rate	Log_2_(OR)
rs35749011	1	155,135,036	*GBA – SYT11*	A	0.017	6.09 × 10^−23^	2.307	0.021	0.999	1.206
rs823118	1	205,723,572	*RAB7L1 – NUCKS1*	T	0.559	1.36 × 10^−13^	1.109	0.412	0.945	0.149
rs10797576	1	232,664,611	*SIPA1L2*	T	0.140	1.19 × 10^−08^	1.110	0.142	0.125	*not included*
rs6430538	2	135,539,967	*ACMSD – TMEM163*	T	0.430	5.56 × 10^−15^	0.882	0.430	0.998	−0.181
rs1474055	2	169,110,394	*STK39*	T	0.128	7.12 × 10^−16^	1.218	0.133	0.991	0.285
rs115185635	3	87,520,857	*KRT8P25 – APOOP2*	C	0.035	2.18 × 10^−08^	0.931	0.035	0.123	*not included*
rs12637471	3	182,762,437	*MCCC1*	A	0.193	3.32 × 10^−16^	0.836	0.183	1.000	−0.258
rs34311866	4	951,947	*TMEM175 – GAK - SGKQ*	T	0.809	3.58 × 10^−33^	0.791	0.779	0.998	−0.338
rs11724635	4	15,737,101	*BST1*	A	0.553	8.07 × 10^−13^	1.138	0.439	1.000	0.187
rs6812193	4	77,198,986	*FAM47E – SCARB2*	T	0.364	7.17 × 10^−11^	0.935	0.347	1.000	−0.097
rs356182	4	90,626,111	*SNCA*	A	0.633	3.23 × 10^−67^	0.822	0.599	0.872	−0.085
rs9275326	6	32,666,660	*HLA – DQB1*	T	0.094	5.82 × 10^−13^	0.900	0.100	0.995	−0.152
rs199347	7	23,293,746	*GPNMB*	A	0.590	2.37 × 10^−12^	1.072	0.595	0.996	0.100
rs117896735	10	121,536,327	*INPP5F*	A	0.014	1.21 × 10^−11^	1.404	0.004	0.967	0.147
rs3793947	11	83,544,472	*DLG2*	A	0.443	2.59 × 10^−08^	0.976	–	–	*not available*
rs329648	11	133,765,367	*MIR4697*	T	0.354	1.65 × 10^−08^	0.121	0.359	0.126	*not included*
rs76904798	12	40,614,434	*LRRK2*	T	0.143	1.33 × 10^−12^	1.110	0.151	0.999	0.151
rs11060180	12	123,303,586	*CCDC62*	A	0.558	2.14 × 10^−08^	1.114	0.560	0.872	0.156
rs11158026	14	55,348,869	*GCH1*	T	0.335	7.13 × 10^−11^	0.889	0.314	0.123	*not included*
rs1555399	14	67,984,370	*TMEM229B*	A	0.468	5.53 × 10^−16^	0.872	0.509	0.125	*not included*
rs2414739	15	61,994,134	*VPS13C*	A	0.734	4.13 × 10^−09^	1.114	0.781	0.127	*not included*
rs14235	16	31,121,793	*BCKDK – STX1B*	A	0.381	3.89 × 10^−08^	1.094	0.391	0.995	0.130
rs17649553	17	43,994,648	*MAPT*	T	0.226	4.86 × 10^−37^	0.771	0.201	0.991	−0.113
rs12456592	18	40,673,380	*RIT2*	A	0.693	5.12 × 10^−09^	0.905	0.756	0.126	*not included*
rs62120679	19	2,363,319	*SPPL2B*	T	0.314	2.53 × 10^−09^	1.141	–	–	*not available*
rs8118008	20	3,168,166	*DDRGK1*	A	0.657	2.32 × 10^−08^	1.111	0.609	0.126	*not included*

### Statistical analysis

The effect and statistical significance of the PRS with PD status was calculated using general linear models (The R Foundation for Statistical Computing). The ROC curve was calculated using the R package pROC [38]. The CSF biomarker levels were normalized and standardized to zero to account for the different platforms used in the cohorts [39]. Briefly, CSF biomarker levels were log_10_-tranformed to normalize the distribution of the values; then, the mean from each dataset was used to standardize to zero. Finally the possible association between CSF biomarker levels and the PRS was tested using general linear models. All models were adjusted by age (at last assessment for PD status and at lumbar puncture (LP) for the CSF biomarker levels), sex and population admixture as represented by the first two principal components in all of the analyses. The association analysis with age at onset was performed using a survival analysis with the R package Survival, using Cox regression. Tertiles of the PRS were calculated and used to perform a Kaplan-Meier analysis and to estimate the effect (OR) between the first and third tertiles. In both cases, age at onset for PD cases was used as the event and age at last assessment was censored for controls. We also performed the same analyses splitting the PD population by existence of family history of PD to assess if the effect of the PRS was different in the two subsets. The theoretical maximum of the calculated PRS is 2.5 and the minimum −2.1, and the beta for each PRS analyses are expressed per unit of PRS.

## Results

### Parkinson disease risk

**Fig. 1:**
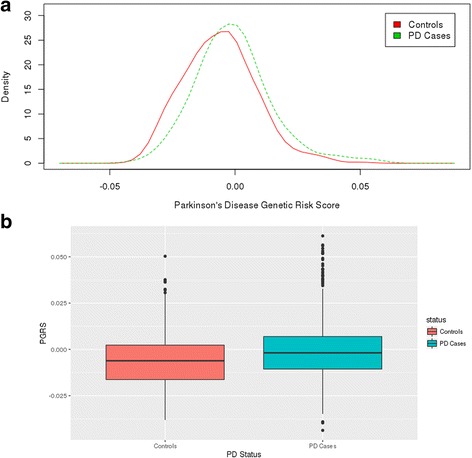
Genetic Risk Score distribution between cases and controls. a PRS distribution by PD status. The line represents the controls and the dotted line represents the PD cases. b PRS box plots by PD status. Case contol status is indicated in the x axis

The PRS was significantly associated with PD status in the joint analysis (*p* = 5.83×10^−08^, beta = 5.24), as well as in each individual dataset [PPMI (*p* = 3.45×10^−05^, beta = 5.84); WUSTL (*p* = 1.82×10^−04^, beta = 4.85)] (Table 3, Fig. 1 Panels A and B). Among the variants that form the PRS, three were nominally associated with PD status in the combined dataset (rs12637471 (*MCCC1*), rs34311866 (*TMEM170-GAK-SGKQ*) and rs356182 (*SNCA*) with *p*-values that range between 4.13 × 10^−04^ and 0.01 (Additional file 1: Table S1). One variant also showed a trend toward association (rs1474055 (*STK39*) (Additional file 1: Table S1).

**Table 3:** Association between Genetic Risk Score and PD Status, age at onset and CSF Biomarker Levels

Trait^a^	Combined			PPMI			WUSTL		
	*P*.Value	Beta^b^	95% CI	*P*.Value	Beta^b^	95% CI	*P*.Value	Beta^b^	95% CI
PD Status	**5.83 × 10** ^**−08**^	5.24	3.35–7.12	**3.45 × 10** ^**−05**^	5.84	3.10–8.59	**1.82 × 10** ^**−04**^	4.85	2.32–7.39
Age at Onset (Survival)	**5.70 × 10** ^**−07**^	11.20	6.81–15.58	**3.19 × 10** ^**−06**^	16.62	9.63–23.61	**1.41 × 10** ^**−03**^	9.30	3.59–15.00
CSF α-Syn Levels	0.21	−0.57	−1.47 – 0.33	0.66	−0.24	−1.30 – 0.82	0.06	−1.60	−3.28 – 0.07
CSF Aβ_1–42_ Levels	0.06	−0.63	−1.29 – 0.03	0.27	−0.44	−1.21 - -0.33	0.07	−1.19	−2.50 – 0.11
CSF Tau Levels	**0.02**	−1.01	−1.87 - -0.16	0.06	−0.95	−1.94 – 0.04	0.17	−1.20	−2.95 – 0.54
CSF P-Tau Levels	0.15	−0.87	−2.07 – 0.33	0.21	−0.87	−2.23 – 0.50	0.31	−1.13	−3.33 – 1.06

^a^Statistical model for each trait: PD-Status ~ PRS + age at last assessment + sex + PC1 + PC2; Age at Onset ~ PRS + sex + PC1 + PC2 (Cox Regression); α-synuclein levels ~ PRS + age at LP + sex + PC1 + PC2; Aβ_1–42_ levels ~ PRS + age at LP + sex + PC1 + PC2; Tau levels ~ PRS + age at LP + sex + PC1 + PC2; P-Tau levels ~ PRS + age at LP + sex + PC1 + PC2

^b^Beta per PRS unit In bold are the statistically significant *p* values

The most significant variant in the joint analysis was rs356182, located on the *SNCA* gene region (*p* = 4.13 × 10^−04^). To determine if the association of the PRS was driven by the *SNCA* variant, we calculated and re-tested a PRS without the SNCA variant in the joint-analysis. The PRS without the SNCA variant showed a similar *p*-value and effect size (*p* = 5.14 × 10^−07^, beta = 4.60) to that of the full PRS. This result suggests that the association was not driven by the *SNCA* variant. These results also suggest that the PRS provide much more statistical power than the single variant analysis.

The analyses in the subsets with and without family history of PD yield similar results. The PRS was associated with PD risk in both subsets (family history: *p* = 5.90 × 10^−06^, beta = 6.13; no family history: *p* = 1.80×10^−06^, beta = 5.08).

### Parkinson disease age at onset

**Fig. 2:**
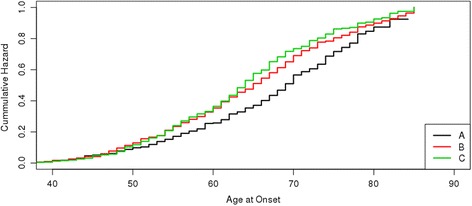
Kaplan-Meier Survival and Cumulative Hazards curves for Age at Onset for PD-PRS by Tertiles. A-1st Tertile (black line), B-2nd Tertile (red line), C-3rd Tertile (green line)

To ascertain the effect of the PRS on age at onset, we applied a Cox survival model. Higher PRS was significantly associated with earlier age at onset (*p* = 5.70 × 10^−07^, beta = 11.20; OR_estimate_ = 4.99; based on a Log-Rank test for the first and third tertiles comparison; see Material and Methods) (Table 3 and Fig. 2), suggesting that variants in the PRS have an additive effect on age at onset. To ascertain for an artifactual result due to control censoring, we tested the survival model using only PD cases. In this sensitivity analysis, the model remained significant (*p* = 0.01) and the effect size had the same direction and comparable effect size (beta = 5.83; OR_estimate_ = 4.91; based on a Log-Rank test for the first and third tertiles comparison; see Material and Methods).

The most significant variant associated with age at onset was located in the *GBA* gene (rs35749011: *p* = 5.00 × 10^−03^, OR = 1.57) (Additional file 1: Table S2). The PRS was still associated with age at onset with similar effect when removing this variant (*p* = 5.60×10^−05^, beta = 9.83; OR_estimate_ = 3.79; based on a Log-Rank test for the first and third tertiles comparison; see Material and Methods). This variant was not associated with PD risk in our analyses (Additional file 1: Table S1), suggesting that the effect on age at onset is greater than the effect on PD risk. The variants in the *MCC1, TMEM170-GAK-SGKQ* and *SNCA* gene regions were associated with both age at onset and PD risk and had similar effect sizes (Additional file 1: Tables S1 and S2). This result suggests that these genes may be affecting multiple aspects of the disease at the same time.

The effect of the PRS was associated with age at onset in both cases without family history (*p* = 2.57×10^−06^, beta = 11.94, OR_estimate_ = 6.53) and with family history (*p* = 2.50×10^−05^, beta = 19.50, OR_estimate_ = 4.84).

### CSF biomarker levels

To test if the genetic architecture of PD risk and that of CSF α-syn, Aβ_1–42_, t-tau or p-tau levels have some overlap, we tested for association between the PRS for PD risk and CSF biomarker levels (*N* = 577). The PRS for PD risk was nominally associated with t-tau in the expected direction (*p* = 0.02, OR = 0.36; higher PRS, lower CSF tau) but not with α-syn (*p* = 0.20, OR = 0.57), Aβ_1–42_ (*p* = 0.05, OR = 0.52; higher PRS, lower CSF Aβ_1–42_) or p-tau (*p* = 0.11, OR = 0.39) levels, although all of the ORs were also in the expected direction (Table 3). Only CSF tau levels were found nominally associated with the PRS in the cases with family history (*p* = 0.04, OR = 0.25), but not in the ones without. No other CSF analyte was found associated with the PRS in these sub-analyses.

None of the variants were found to be associated with CSF t-tau levels. Only the variant rs34311866, located in the *TMEM170-GAK-SGKQ* gene region, was nominally associated with CSF t-tau levels in the WUSTL dataset (*p* = 0.03, OR = 2.59) (Additional file 1: Table S3). The variant in *MAPT* rs17649553 was not associated with t-tau or p-tau levels. No additional variants were found associated with CSF t-tau levels, suggesting that the effect of the PRS variants on CSF t-tau levels is additive. For CSF Aβ_1–42_ one variant was statistically significant in the combined dataset (rs6812193: *p* = 2.58×10^−03^, OR = 1.02) (Additional file 1: Table S4). The variant located in the *LRKK2* gene (a known Mendelian PD gene [26]) was found to be nominally associated with Aβ_1–42_ levels (p = 0.04, OR = 0.96) and to CSF α-syn levels (*p* = 9.31×10^−04^, OR = 0.92) in the WUSTL dataset, but not in the PPMI dataset or the combined dataset. No Any other variant was found associated with α-syn (Additional file 1: Table S5) or p-tau CSF levels (Additional file 1: Table S6).

## Discussion

This study aimed to test if the known genetic variants associated with PD risk have a cumulative effect on PD risk, age at onset or CSF biomarker levels. We calculated a weighted PRS using previously reported GWAs loci [7]. Even though we were not covering all of the genetic architecture of PD (due to the inclusion of GWAs hits only), the PRS was associated with PD status and age at onset when using a survival model. In regard to the CSF biomarkers, the PRS was only nominally associated with t-tau levels.

Even though the PRS was constructed with known genome-wide loci [7], not all of the variants were associated with PD risk in our analyses. This suggests that our study could be underpowered for the replication of all the known loci at the single variant level. Nevertheless, the statistical significance of the PRS shows that the reported variants associated with PD risk have a cumulative effect on the PD risk and provide more power than the single variant analyses even when the most significant variant was removed. Here we describe a very strong association of the PRS with PD risk (*p* = 5.83×10^−08^, beta = 5.24) even though we were only able to include 16 of the 26 variants. Moreover, this suggests that the PRS provides more robust results than single variant analyses and, according to our results, independently of family history. Our results show that the PRS effect is similar in PD cases with and without family history. In conclusion, it is plausible to think that the genetic architecture of idiopathic and familial PD is to some extent shared.

The advantage of the PRS over single variant analyses is that it aggregates the additive effect (in both directions, protective and risk) of several variants with small effect individually [22]. A perfect PRS will allow the capture or summary of all the genetic architecture of a disease in one value or clinical test. As a result, a PD-PRS will be more useful in the idiopathic PD setting due to the unknown cause of the disease. The PRS was associated with cases with and without family history in our dataset. This dual association increases the possibility of the use of an improved PRS as a clinical tool in the future due to its strength in capturing the cumulative genetic variation.

When the PRS was added to PD risk score modeled with age, sex and the first two principal components, the AUC improved about three percent (data not shown). Even though this improvement is modest, it is likely that future studies including additional GWAs loci will improve the predictive value of the PRS. For example, in schizophrenia studies, the PRS improved one and a half fold when any loci with a *p*-value lower than 0.1 was included [20]. Future analyses focused on generating PRS for PD should include an analysis of what is the most informative inclusion threshold to create the most predictive PRS.

In this study we have been able to replicate the association of the PD risk loci by using a PRS approach and furthermore have replicated an association of the PRS with age at onset [10]. For the age at onset analyses we used a survival model because it provides more power than a simple linear regression. In our datasets we found that *SNCA* and *GBA* variants have the strongest effect in risk and age at onset respectively, supporting previous studies [40]. Low frequency mutations with large effect sizes have been previously reported in known PD genes such as *LRRK2, PARK2* or *SNCA.* These variants are known to cause PD and have been reported to reduce age at onset [31, 41]. Therefore, additional studies should be performed to determine whether or not the inclusion of these variants would strengthen PRS calculations. In any case, our results suggest that the genetic architecture of age at onset is more complex with contributions from known variants and potentially many others.

Recent studies have shown that CSF levels of α-syn, t-tau, p-tau or Aβ_1–42_ are lower in individuals with PD [14, 19]. Therefore, we wanted to test if the genetic architecture of PD risk related to CSF levels of these four biomarkers. Previous studies have shown that disease risk PRS was associated with disease biomarkers. For example, a PRS calculated based on GWAS hits for Alzheimer’s Disease was strongly associated with CSF Aβ_1–42_ and t-tau levels (*p* = 5.01 × 10^−7^ and *p* = 1.81 × 10^−8^; respectively) [39]. We hypothesized that the PD risk PRS will similarly be associated with CSF levels of the relevant proteins. However, our results did not support this hypothesis in our dataset. The lack of association between the PRS and CSF α-syn levels is probably due to lack of power or a potential biological difference in the relationship between CSF α-syn levels and PD risk. Interestingly, we have found the PD-PRS marginally related to CSF t-tau levels. The variant included in the PD-PRS from the *MAPT* gene (rs17649553), a known expression quantitative trait loci (eQTL) for MAPT [42] was not significantly associated with t-tau levels as previously described [31]. Consequently, this association is probably due to the genetic load of PD risk alleles. Finally, we have also found a trend towards association with Aβ_1–42_ levels. This effect agrees with the previous findings [14, 19] of higher PD genetic load and lower Aβ_1–42_ levels in PD patients. However, larger studies are needed to demonstrate if this trend is a true association.

The main limitation of this study is the use of common genome-wide significant and replicated variants to evaluate the genetic overlap between disease risk, age at onset and CSD biomarker levels. A recent study indicates that the SNPs that are significant for disease risk but do not pass the multiple test correction of GWA studies, can still be informative for the PRS [9]. Other studies show that low frequency variants, not analyzed in GWA studies are also associated with disease risk [43, 44]. Therefore, further studies in PD (including common and rare variants) may provide a more accurate estimation of the genetic overlap among the different PD characteristics.

## Conclusions

In conclusion, the known genetic architecture of PD risk has cumulative effects on PD risk and age at onset. However, this genetic signature does not seem to be related to CSF levels of α-synuclein but does relate to t-tau levels. Even though many GWAs loci have been found in previous studies, additional analyses with larger sample sizes are needed to elucidate the still missing heritability of PD and to create a clinically useful PRS.

## Additional file

Additional file 1: Tables S1 – S6. (DOCX 38 kb)

## Abbreviations

AD: Alzheimer’s disease
Aβ_1–42_: Amyloid beta 1–42
CBD: Corticobasal degeneration
CSF: Cerebrospinal fluid
GWAs: Genome-wide association studies
HWE: Hardy Weinberg equilibrium
LP: Lumbar puncture
MAF: Minor allele frequency
PC: Principal component
PD: Parkinson ’s disease
PPMI: Parkinson’s Progressive Markers Initiative
PRS: Polygenic risk score
PSP: Progressive supranuclear palsy
p-tau: Phosphorilated tau
SNCA: Alpha-synuclein gene
t-tau: Total tau
WUSTL: Washington University in Saint Louis
