# Supplementary material for: Parkinson disease polygenic risk score is associated with Parkinson disease status and age at onset but not with alpha-synuclein cerebrospinal fluid levels
Source: BMC Neurol. 2017 Nov 15;17:198. doi: 10.1186/s12883-017-0978-z (PMC5688622; doi:10.1186/s12883-017-0978-z)
Supplement: Additional file 1: — Tables S1 – S6. (DOCX 38 kb) [file 12883_2017_978_MOESM1_ESM.docx]

**SUPPLEMENTARY DATA**

**Supplementary Table 1.** PD Genetic Risk Score variants summary for PD Status

| **Variant** | **Gene or Nearest Gene** | **Allele** | **Combined** | | **PPMI** | | **WUSTL** | |
| --- | --- | --- | --- | --- | --- | --- | --- | --- |
|  |  |  | **P.Value** | **OR** | **P.Value** | **OR** | **P.Value** | **OR** |
| rs35749011 | *GBA-SYT11* | A | 0.16 | 1.62 | 0.23 | 2.15 | 0.52 | 1.35 |
| rs823118 | *RAB7L1-NUCKS1* | T | 0.22 | 0.89 | 0.74 | 1.05 | 0.08 | 0.83 |
| rs6430538 | *ACMD-TMEM163* | T | 0.38 | 0.93 | 0.43 | 0.89 | 0.55 | 0.90 |
| rs1474055 | *STK39* | T | 0.05 | 1.30 | 0.54 | 1.14 | 0.05 | 1.31 |
| rs12637471 | *MCCC1* | A | **0.01** | 0.73 | 0.10 | 0.74 | **0.02** | 0.81 |
| rs34311866 | *TMEM170-GAK-SGKQ* | T | **0.03** | 1.27 | 0.05 | 1.42 | 0.30 | 1.18 |
| rs11724635 | *BST1* | A | 0.40 | 0.93 | 0.04 | 0.75 | 0.77 | 1.09 |
| rs6812193 | *FAM47E-SCARB2* | T | 0.32 | 0.91 | 0.77 | 0.96 | 0.15 | 0.88 |
| rs356182 | *SNCA* | A | **4.13×10^-04^** | 1.39 | **0.02** | 1.42 | **5.20×10^-03^** | 1.44 |
| rs9275326 | *HLA-DQB1* | T | 0.19 | 0.82 | **0.01** | 0.56 | 0.55 | 1.10 |
| rs199347 | *GPNMB* | A | 0.11 | 0.86 | 0.38 | 0.88 | 0.22 | 0.80 |
| rs117896735 | *INPP5F* | A | 0.15 | 0.19 | 0.58 | 0.46 | NA | NA |
| rs76904798 | *LRRK2* | T | 0.11 | 1.23 | **0.03** | 1.71 | 0.55 | 1.21 |
| rs11060180 | *CCDC62* | A | 0.51 | 0.94 | 0.59 | 0.93 | 0.51 | 0.88 |
| rs14235 | *BCKDK-STX1B* | A | 0.06 | 1.19 | 0.60 | 1.08 | 0.07 | 1.30 |
| rs17649553 | *MAPT* | T | 0.73 | 1.04 | 0.99 | 1.00 | 0.99 | 1.07 |

In bold the statistically significant p values

**Supplementary Table 2.** PD Genetic Risk Score variants summary for Age at Onset (Survival)

| **Variant** | **Gene or Nearest Gene** | **Allele** | **Combined** | | **PPMI** | | **WUSTL** | |
| --- | --- | --- | --- | --- | --- | --- | --- | --- |
|  |  |  | **P.Value** | **OR^a^** | **P.Value** | **OR^a^** | **P.Value** | **OR^a^** |
| rs35749011 | *GBA-SYT11* | A | **5.00×10^-03^** | 1.57 | **0.02** | 1.81 | 0.05 | 1.48 |
| rs823118 | *RAB7L1-NUCKS1* | C | 0.22 | 0.94 | 0.20 | 0.90 | 0.43 | 0.95 |
| rs6430538 | *ACMD-TMEM163* | T | 0.76 | 1.02 | 0.69 | 1.03 | 1.00 | 1.00 |
| rs1474055 | *STK39* | T | 0.17 | 1.10 | 0.98 | 1.00 | 0.10 | 1.16 |
| rs12637471 | *MCCC1* | A | **0.02** | 0.86 | 0.30 | 0.90 | 0.05 | 0.85 |
| rs34311866 | *TMEM170-GAK-SGKQ* | C | **0.01** | 1.16 | **5.20×10^-03^** | 1.30 | 0.20 | 1.10 |
| rs11724635 | *BST1* | C | 0.17 | 0.93 | 0.05 | 0.85 | 0.57 | 0.96 |
| rs6812193 | *FAM47E-SCARB2* | T | 0.15 | 0.93 | 0.27 | 0.91 | 0.17 | 0.91 |
| rs356182 | *SNCA* | C | **0.01** | 1.14 | 0.06 | 1.16 | 0.06 | 1.15 |
| rs9275326 | *HLA-DQB1* | T | 0.92 | 0.99 | **0.02** | 0.70 | 0.07 | 1.21 |
| rs199347 | *GPNMB* | G | 0.07 | 0.91 | 0.17 | 0.89 | 0.30 | 0.93 |
| rs117896735 | *INPP5F* | A | 0.42 | 1.39 | 0.31 | 2.77 | 0.44 | 1.41 |
| rs76904798 | *LRRK2* | T | 0.31 | 1.07 | **0.05** | 1.26 | 0.91 | 1.01 |
| rs11060180 | *CCDC62* | G | 0.73 | 1.02 | 0.94 | 0.99 | 0.97 | 1.00 |
| rs14235 | *BCKDK-STX1B* | A | 0.25 | 1.06 | 0.81 | 1.01 | 0.19 | 1.09 |
| rs17649553 | *MAPT* | T | 0.06 | 0.89 | 0.53 | 0.95 | 0.06 | 0.58 |

^a^Odds Ratio calculated with e to the beta from the cox regression model

In bold the statistically significant p values

**Supplementary Table 3.** PD Genetic Risk Score variants summary for Tau

| **Variant** | **Gene or Nearest Gene** | **Allele** | **Combined** | | **PPMI** | | **WUSTL** | |
| --- | --- | --- | --- | --- | --- | --- | --- | --- |
|  |  |  | **P.Value** | **OR^a^** | **P.Value** | **OR^a^** | **P.Value** | **OR^a^** |
| rs35749011 | *GBA-SYT11* | A | 0.36 | 0.97 | 0.12 | 0.94 | 0.37 | 1.05 |
| rs823118 | *RAB7L1-NUCKS1* | C | 0.05 | 1.02 | 0.09 | 1.02 | 0.18 | 1.03 |
| rs6430538 | *ACMD-TMEM163* | T | 0.09 | 1.02 | 0.23 | 1.01 | 0.37 | 1.02 |
| rs1474055 | *STK39* | T | 0.60 | 0.99 | 0.34 | 0.99 | 0.60 | 1.02 |
| rs12637471 | *MCCC1* | A | 0.40 | 1.01 | 0.39 | 1.01 | 0.94 | 1.00 |
| rs34311866 | *TMEM170-GAK-SGKQ* | C | 0.05 | 0.98 | 0.25 | 0.99 | **0.03** | 0.95 |
| rs11724635 | *BST1* | C | 0.56 | 0.99 | 0.58 | 0.99 | 0.99 | 1.00 |
| rs6812193 | *FAM47E-SCARB2* | T | 0.05 | 1.02 | 0.06 | 1.02 | 0.76 | 1.01 |
| rs356182 | *SNCA* | C | 0.16 | 1.02 | 0.11 | 1.02 | Monomorphic | |
| rs9275326 | *HLA-DQB1* | T | 0.29 | 0.98 | 0.40 | 0.98 | 0.37 | 0.97 |
| rs199347 | *GPNMB* | G | 0.87 | 1.00 | 1.00 | 1.00 | 0.79 | 1.01 |
| rs117896735 | *INPP5F* | A | 0.79 | 0.98 | 0.89 | 0.98 | 0.84 | 0.98 |
| rs76904798 | *LRRK2* | T | 0.76 | 1.00 | 0.28 | 1.02 | 0.06 | 0.95 |
| rs11060180 | *CCDC62* | G | 0.74 | 1.00 | 0.83 | 1.00 | Monomorphic | |
| rs14235 | *BCKDK-STX1B* | A | 0.81 | 1.00 | 0.19 | 1.01 | 0.06 | 0.96 |
| rs17649553 | *MAPT* | T | 0.74 | 1.00 | 0.81 | 1.00 | 0.85 | 1.01 |

^a^Odds Ratio calculated with e^beta^ from the linear model

In bold the statistically significant p values

**Supplementary Table 4.** PD Genetic Risk Score variants summary for A-beta

| **Variant** | **Gene or Nearest Gene** | **Allele** | **Combined** | | **PPMI** | | **WUSTL** | |
| --- | --- | --- | --- | --- | --- | --- | --- | --- |
|  |  |  | **P.Value** | **OR^a^** | **P.Value** | **OR^a^** | **P.Value** | **OR^a^** |
| rs35749011 | *GBA-SYT11* | A | 0.38 | 0.98 | 0.27 | 0.97 | 0.96 | 1.00 |
| rs823118 | *RAB7L1-NUCKS1* | C | 0.44 | 1.01 | 0.61 | 1.00 | 0.45 | 1.01 |
| rs6430538 | *ACMD-TMEM163* | T | 0.44 | 1.01 | 0.81 | 1.00 | 0.13 | 1.03 |
| rs1474055 | *STK39* | T | 0.84 | 1.00 | 0.85 | 1.00 | 0.27 | 1.03 |
| rs12637471 | *MCCC1* | A | 0.90 | 1.00 | 0.72 | 1.00 | 0.33 | 1.02 |
| rs34311866 | *TMEM170-GAK-SGKQ* | C | 0.31 | 0.99 | 0.44 | 0.99 | 0.44 | 0.99 |
| rs11724635 | *BST1* | C | 0.59 | 1.00 | 0.80 | 1.00 | 0.43 | 0.99 |
| rs6812193 | *FAM47E-SCARB2* | T | **2.58×10^-03^** | 1.02 | **4.09×10^-03^** | 1.03 | 0.29 | 1.02 |
| rs356182 | *SNCA* | C | 0.32 | 1.01 | 0.34 | 1.01 | Monomorphic | |
| rs9275326 | *HLA-DQB1* | T | 0.24 | 1.01 | 0.40 | 1.01 | 0.40 | 1.02 |
| rs199347 | *GPNMB* | G | 0.46 | 1.01 | 0.97 | 1.00 | 0.14 | 1.02 |
| rs117896735 | *INPP5F* | A | 0.63 | 1.03 | 0.68 | 1.04 | 0.68 | 1.03 |
| rs76904798 | *LRRK2* | T | 0.81 | 1.00 | 0.13 | 1.02 | **0.04** | 0.96 |
| rs11060180 | *CCDC62* | G | 0.28 | 1.01 | 0.27 | 1.01 | Monomorphic | |
| rs14235 | *BCKDK-STX1B* | A | 0.56 | 1.00 | 0.31 | 1.01 | 0.41 | 0.99 |
| rs17649553 | *MAPT* | T | 0.68 | 1.00 | 0.80 | 1.00 | 0.08 | 1.04 |

^a^Odds Ratio calculated with e^beta^ from the linear model

In bold the statistically significant p values

**Supplementary Table 5.** PD Genetic Risk Score variants summary for CSF α-synuclein levels

| **Variant** | **Gene or Nearest Gene** | **Allele** | **Combined** | | **PPMI** | | **WUSTL** | |
| --- | --- | --- | --- | --- | --- | --- | --- | --- |
|  |  |  | **P.Value** | **OR^a^** | **P.Value** | **OR^a^** | **P.Value** | **OR^a^** |
| rs35749011 | *GBA-SYT11* | A | 0.86 | 0.99 | 0.71 | 0.99 | 0.83 | 1.01 |
| rs823118 | *RAB7L1-NUCKS1* | C | 0.12 | 1.02 | 0.24 | 1.01 | 0.19 | 1.03 |
| rs6430538 | *ACMD-TMEM163* | T | 0.92 | 1.00 | 0.86 | 1.00 | 0.78 | 0.99 |
| rs1474055 | *STK39* | T | 0.38 | 0.99 | 0.32 | 0.98 | 0.97 | 1.00 |
| rs12637471 | *MCCC1* | A | 0.52 | 1.01 | 0.80 | 1.00 | 0.32 | 1.03 |
| rs34311866 | *TMEM170-GAK-SGKQ* | C | 0.30 | 0.99 | 0.72 | 1.00 | 0.09 | 0.96 |
| rs11724635 | *BST1* | C | 0.91 | 1.00 | 0.89 | 1.00 | 0.92 | 1.00 |
| rs6812193 | *FAM47E-SCARB2* | T | 0.43 | 1.01 | 0.43 | 1.01 | 0.94 | 1.00 |
| rs356182 | *SNCA* | C | 0.45 | 1.01 | 0.45 | 1.01 | Monomorphic | |
| rs9275326 | *HLA-DQB1* | T | 0.26 | 0.98 | 0.39 | 0.98 | 0.48 | 0.98 |
| rs199347 | *GPNMB* | G | 0.93 | 1.00 | 0.97 | 1.00 | 0.84 | 1.00 |
| rs117896735 | *INPP5F* | A | 0.68 | 0.97 | 0.98 | 1.00 | 0.67 | 0.96 |
| rs76904798 | *LRRK2* | T | 0.30 | 0.99 | 0.57 | 1.01 | **9.31×10^-04^** | 0.92 |
| rs11060180 | *CCDC62* | G | 0.71 | 1.00 | 0.71 | 1.00 | Monomorphic | |
| rs14235 | *BCKDK-STX1B* | A | 0.74 | 1.00 | 0.23 | 1.01 | 0.06 | 0.96 |
| rs17649553 | *MAPT* | T | 0.81 | 1.00 | 0.96 | 1.00 | 0.60 | 1.02 |

^a^Odds Ratio calculated with e^beta^ from the linear model

In bold the statistically significant p values

**Supplementary Table 6.** PD Genetic Risk Score variants summary for p tau

| **Variant** | **Gene or Nearest Gene** | **Allele** | **Combined** | | **PPMI** | | **WUSTL** | |
| --- | --- | --- | --- | --- | --- | --- | --- | --- |
|  |  |  | **P.Value** | **OR^a^** | **P.Value** | **OR^a^** | **P.Value** | **OR^a^** |
| rs35749011 | *GBA-SYT11* | A | 0.92 | 1.00 | 0.98 | 1.00 | 0.66 | 1.03 |
| rs823118 | *RAB7L1-NUCKS1* | C | 0.59 | 1.01 | 0.84 | 1.00 | 0.18 | 1.04 |
| rs6430538 | *ACMD-TMEM163* | T | 0.07 | 1.02 | 0.21 | 1.02 | 0.08 | 1.05 |
| rs1474055 | *STK39* | T | 0.35 | 0.98 | 0.19 | 0.97 | 0.34 | 1.04 |
| rs12637471 | *MCCC1* | A | 0.69 | 0.99 | 0.87 | 1.00 | 0.65 | 0.98 |
| rs34311866 | *TMEM170-GAK-SGKQ* | C | 0.06 | 0.97 | 0.09 | 0.97 | 0.15 | 0.96 |
| rs11724635 | *BST1* | C | 0.84 | 1.00 | 0.96 | 1.00 | 0.81 | 0.99 |
| rs6812193 | *FAM47E-SCARB2* | T | 0.65 | 1.01 | 0.70 | 1.01 | 0.69 | 1.01 |
| rs356182 | *SNCA* | C | 0.26 | 1.02 | 0.25 | 1.02 | Monomorphic | |
| rs9275326 | *HLA-DQB1* | T | 0.26 | 0.98 | 0.41 | 0.98 | 0.17 | 0.94 |
| rs199347 | *GPNMB* | G | 0.39 | 1.01 | 0.46 | 1.01 | 0.97 | 1.00 |
| rs117896735 | *INPP5F* | A | 0.44 | 0.92 | 0.29 | 0.85 | 0.63 | 0.94 |
| rs76904798 | *LRRK2* | T | 0.83 | 1.00 | 0.67 | 1.01 | 0.08 | 0.94 |
| rs11060180 | *CCDC62* | G | 0.64 | 1.01 | 0.62 | 1.01 | Monomorphic | |
| rs14235 | *BCKDK-STX1B* | A | 0.49 | 1.01 | 0.28 | 1.02 | 0.43 | 0.98 |
| rs17649553 | *MAPT* | T | 0.98 | 1.00 | 0.99 | 1.00 | 0.77 | 1.01 |

^a^Odds Ratio calculated with e^beta^ from the linear model

In bold the statistically significant p values
